# 11989 The Impact of a Perinatal Mental Health Clinic on Psychopathology

**DOI:** 10.1017/cts.2021.582

**Published:** 2021-03-30

**Authors:** Danielle Cooke

**Affiliations:** University of Florida

## Abstract

ABSTRACT IMPACT: This research is intended to provide researchers and clinicians information on factors that impact psychiatric health outcomes in a specialty perinatal mood disorders clinic. OBJECTIVES/GOALS: The present study seeks to examine factors that impact psychiatric outcomes at the University of Florida Department of Obstetrics and Gynecology Perinatal Mood Disorders Clinic (PMDC). METHODS/STUDY POPULATION: A hierarchical multinomial logistic regression will be conducted to evaluate predictors that may influence patients receiving a referral to specialty care, a return to primary care or being lost to follow up. Included predictors are changes in insurance status, baseline depression scores, and baseline obsessive-compulsive symptoms (OCS). A multinomial logistic regression will be conducted to determine if OCS and depressive symptoms predict referral to/establishment of psychotherapeutic care. A secondary binary logistic regression will be conducted to evaluate predictors that may predict reduction in depressive symptoms among women seen for more than one session. Included predictors of outcome include time (weeks in psychiatric treatment), OCS at baseline, and referral to psychological therapy. RESULTS/ANTICIPATED RESULTS: Data collection is multiphasic and ongoing via a retrospective chart review of patients seen in the PMDC. Hypotheses include that experiencing a change in insurance will significantly increase the risk of being lost to follow up, as compared to referral to specialty clinic or returning to primary care. It is also predicted that individuals with higher depressive symptoms or OCS will be more likely likely to be assigned to specialty care than to be lost to follow up or primary care. It is believed that greater time in psychiatric care, and lower OCS will increase the likelihood of reductions in depressive symptoms. DISCUSSION/SIGNIFICANCE OF FINDINGS: This study seeks to provide information on predictors that influence outcome this specialty clinic, while extending the limited literature that has examined the influence of OCS on depressive symptoms. It is the hope of the authors to provide information on intervenable factors that influence psychiatric outcomes in a perinatal specialty clinic.

